# 

**Published:** 1884-08

**Authors:** 


					﻿(Whole Number,
CCLXVII.
AUGUST, 1884.
Number i,
VotfjMEj Xxiy.
THE
BU FFALO
MediGal and Surgical Journal.
EDITORS :
THOS. LOTHROP, M.
A. R. DAVIDSON, M.
Published by the Medical Journal Association.
No. 8 WEST CHIPPEWA ST.
Entered at the Post-Office at Buffalo, N. Y., as Second-class Mail Matter.
				

